# Intensive speech-language pathology therapy with adults who stutter: preliminary study

**DOI:** 10.1590/2317-1782/20232021159en

**Published:** 2023-05-26

**Authors:** Amanda Caroline Pereira da Silva Miranda, Camila Queiroz de Moraes Silveira Di Ninno, Denise Brandão de Oliveira e Britto

**Affiliations:** 1 Departamento de Fonoaudiologia, Faculdade de Medicina, Universidade Federal de Minas Gerais - UFMG - Belo Horizonte (MG), Brasil.; 2 Fonoaudióloga Clínica, Consultório Particular - São Paulo (SP), Brasil.

**Keywords:** Speech Therapy, Language, Language Disorders, Childhood-Onset Fluency Disorder, Stuttering

## Abstract

**Purpose:**

To compare the results of fluency and self-perception of the impact of stuttering on the lives of adults who stutter, before and after undergoing intensive speech-language pathology therapy.

**Methods:**

This is a descriptive and longitudinal study with data collection before and after intensive therapy in four patients who stutter. The intensive care program consisted of thirty one-hour sessions held in five individual sessions a week. Speech samples collected before and after therapy were analyzed by two fluency experts. Descriptive data analysis was performed through the frequency distribution of categorical variables and analysis of measures of central tendency and dispersion of continuous variables. The verification of agreement between the evaluations carried out by the two judges was performed using the intraclass correlation coefficient (ICC). Correlation analysis was also performed using Spearman's rank correlation coefficient between the variables in the speech sample and the OASES-A scores.

**Results:**

There was a reduction of the percentage of stuttering disfluencies, increasing the flow of words per minute of the participants. The descriptive analysis of the OASES-A showed a decrease in the degree of impact of stuttering on the participants' lives in all parts of the questionnaire.

**Conclusion:**

There was an improvement in all variables analyzed after intensive care, including an improvement in speech fluency and a reduction in the impact of stuttering on the participants' lives, which suggests the relevance of the intensive speech therapy proposal for stuttering.

## INTRODUCTION

Developmental stuttering is a fluency disorder characterized by the presence of involuntary interruptions in the speech flow, such as repetitions of sounds, syllables and monosyllabic words, prolongations of sounds, blocks, extensive pauses and intrusions that interrupt the smooth and continuous speech flow^(1)^. Stuttering can be understood as a result of a central nervous system dysfunction, with a genetic origin, which appears in the period of language development, between 18 months and seven years of age. The disorder becomes chronic in 20% of cases, which means a prevalence of 1% in adulthood, with a higher occurrence in males^(2)^.

Stuttering is a developmental disorder with a multidimensional aspect, which can be impacted by several factors, such as family history, environment, linguistic and cognitive abilities of the individual^(3)^. Although the etiology of stuttering has not yet been precisely identified, some studies^(2,3)^ have shown that genetic factors are related to susceptibility to the disorder.

Speech-language pathology intervention is essential in individuals who stutter, as the treatment aims to promote fluency and reduce disfluencies, providing a greater flow of information and continuous and smooth speech, as natural as possible, for both the speaker and the listener^(4)^.

Although speech-language pathology therapy is traditionally performed with one to two sessions per week, there are proposals^(5-7)^ that claim that intensive programs are an alternative to achieve a more fluent speech pattern in less time. In this sense, the proposal of intensive therapy for fluency is based on the North American, Canadian and European models of therapeutic programs for the stimulation of oral language, speech and/or fluency, and is characterized by daily sessions, individual or in groups, for about 30 consecutive days. Given the proximity and frequency of the sessions, it is believed that daily therapy can allow the gradual observation of changes in patients' communicative behavior, helping them to perceive their difficulties and the functional use of language. In addition, intensive care can also be an initial module of conventional therapy or a comprehensive intervention proposal. Generally, speech-language pathology programs for adults, both intensive and conventional, have a mixed approach that involves strategies to promote fluency, modify stuttering and improve communicative skills^(8,9)^.

In this context, this study aimed to compare the results of fluency and self-perception of the impact of stuttering on the lives of adults who stutter before and after undergoing intensive speech-language pathology therapy.

## METHODS

This is a preliminary descriptive and longitudinal study, of an experimental type and of a quantitative nature that analyzed the results of intensive speech-language pathology therapy in four male patients between 20 and 31 years of age, who stutter. The study was approved by the Research Ethics Committee of UFMG, trough n. **CAAE** 02470618.1.0000.5149.

Participants were recruited through an invitation to the population that stutters in Belo Horizonte. The inclusion criteria included the availability to participate in the intensive intervention (five hours/week), the signing of the Informed Consent Form and the presence of a stuttering complaint confirmed by a minimum of 3% of stuttering disfluencies in the analysis of the speech sample^(8)^. In turn, the exclusion criteria were the following: cognitive, psychological and/or neurological impairment, other associated language and neurodevelopmental disorders (self-reported by the participants) and having undergone treatment for stuttering in the last year. The study was carried out at the Speech-Language Pathology Clinic of Hospital São Geraldo, which is part of the Hospital das Clínicas of the UFMG. The following data collection instruments and procedures were applied: Clinical History; Questionnaire about stuttering; Fluency Profile Assessment Protocol (PAPF)^(8)^, Overall Assessment of the Speaker's Experience of Stuttering - Adults (OASES-A), translated into Brazilian Portuguese^(10)^ and an intensive care program for stuttering prepared by the authors based on the literature^(8,9)^.

Initially, the researchers collected the patient's medical history by collecting general information, such as family history of stuttering, history of general health and speech-language disorders, participant's general knowledge of stuttering, main associated factors, onset and severity of stuttering, feelings related to stuttering, impact of stuttering on activities of daily living, and expectations regarding treatment.

Aiming at carrying out the Fluency Profile Assessment Protocol, the researchers obtained the audio and video recording of the participants' spontaneous linked speech sample through the personal presentation and description of a thematic figure, before and after 30 hours of speech-language pathology therapy.

It should be noted that the OASES-A^(10)^ instrument was applied before the start of therapy and at the last therapy session. In this context, the instrument applied is organized into four sections, and each section addresses a different theme, as follows: General Information on Speech, Reaction to Stuttering, Communication in Everyday Situations and Quality of Life. Data were analyzed based on the theoretical frameworks that support the instrument, while the score per section and the global score are obtained by adding the scores of the four sections of the instrument. When interpreting the assessment result, the impact of stuttering on the individual can be understood as mild, mild to moderate, moderate, moderate to severe or severe.

The intensive care program consisted of thirty terapy sessions, which were carried out in five individual sessions a week, totaling a period of approximately two months of consultations. The proposal (Chart 1) was designed based on the speech-language pathology intervention program to promote fluency, with awareness, desensitization and fluency modeling strategies, in addition to activities to modify stuttering and improve communicative skills^(8,9)^.

**Chart 1:** Intervention goals and strategies - Intensive stuttering therapy program

**Sessions**	**Objectives**	**Strategies**
1	● To facilitate voice production	● Body relaxation;
	● To understand how fluent speech and stuttering works (Awareness)	● Breathing exercises;
		● Orofacial motricity exercises;
		● Definition of Stuttering;
		● Actions that affect stuttering positively or negatively;
	● To learn how to make smooth and continuous articulatory contact point (Fluency Modeling)	● Conceptualization of Difficult Speech and Easy Speech;
		● Characteristics of negative speech and practice (100% tension, 50% tension, and smoothed) with a ball: picture naming
2	● To facilitate voice production	● Body relaxation;
	● To understand how fluent speech and stuttering works	● Breathing exercises;
	● To simplify stuttering and get up-to-date scientific information	● Orofacial motricity exercises;
	● To perceive different ways of producing speech (Awareness)	● Dynamics: Speech Machine;
		● Dynamics: Machine failing
		● Myths and Truths about Stuttering
	● To learn how to make and use smooth and continuous articulatory contact point (Fluency Modeling)	● Characteristics of negative speech and practice (100%, 50%, and smoothed) with a ball:
		o Picture naming;
		● Reading word lists looking in the mirror and looking at the therapist.
3	● To facilitate voice production	● Body relaxation;
	● To understand how fluent speech and stuttering works	● Breathing exercises;
	● To perceive different ways of producing speech (Awareness)	● Orofacial motricity exercises;
		● Dynamics: Crazy Speech;
	● To learn how to make and use smooth and continuous articulatory contact point (Fluency Modeling)	● Characteristics of negative speech and practice (100%, 50%, and smoothed) with a ball:
		o Own name;
		o Other names, other naming and *brainstorming*.
4	● To facilitate voice production	● Body relaxation;
	● To understand how fluent speech and stuttering works	● Breathing exercises;
	● To simplify stuttering and get up-to-date scientific information (Awareness)	● Orofacial motricity exercises;
		● Myths and Truths about Stuttering;
	● To know and reduce negative emotions (Desensitization)	● Dynamics: Stuttering Iceberg
	● To learn how to make and use smooth and continuous articulatory contact point (Fluency Modeling)	● Characteristics of negative speech and practice (100%, 50%, and smoothed) with a ball:
		● Other names, other naming and *brainstorming*.
5	● To facilitate voice production	● Body relaxation;
	● To understand how fluent speech and stuttering works	● Breathing exercises;
	● To perceive tension points (Awareness)	● Orofacial motricity exercises;
		● Perception of speech tension points;
	● To learn how to make and use smooth and continuous articulatory contact point (Fluency Modeling)	● Perception of Easy Speech / Normal Speech / Difficult Speech;
		● Characteristics of negative speech and practice (smoothed) with a spring:
		● Repetition of sentences.
6	● To facilitate voice production	● Body relaxation;
	● To understand how fluent speech and stuttering works (Awareness)	● Breathing exercises;
		● Orofacial motricity exercises;
	● To know and reduce negative emotions (Desensitization)	● Good and bad thoughts related to stuttering;
	● To learn and use smooth and continuous articulatory contact point (Fluency Modeling)	● Characteristics of negative speech and practice (smoothed) with a spring:
		o Repetition of sentences;
		o Reading sentences looking in the mirror and looking at the therapist.
7	● To facilitate voice production	● Body relaxation;
	● To understand how fluent speech and stuttering works (Awareness)	● Breathing exercises;
		● Orofacial motricity exercises;
	● To know and reduce negative emotions (Desensitization)	● Role-playing everyday situations;
	● To learn and use smooth and continuous articulatory contact point (Fluency Modeling)	● Characteristics of negative speech and practice (smoothed) with a spring:
		o Reading sentences looking in the mirror and looking at the therapist;
		o Complete ready-made sentences with figures from the deck;
		o Create spontaneous sentences with figures from the deck.
		o Connected speech training with line.
8	● To facilitate voice production	● Body relaxation;
	● To understand how fluent speech and stuttering works (Awareness)	● Breathing exercises;
		● Orofacial motricity exercises;
	● To learn and use smooth and continuous articulatory contact point (Fluency Modeling)	● Characteristics of negative speech and practice (smoothed) with a spring:
		o Complete ready-made sentences with figures from the deck;
		o Create spontaneous sentences with figures from the deck.
		o Role-playing conversations with the line.
9	● To facilitate voice production	● Body relaxation;
	● To understand how fluent speech and stuttering works (Awareness)	● Breathing exercises;
		● Orofacial motricity exercises;
	● To learn and use smooth and continuous articulatory contact point (Fluency Modeling)	● Communicative Skills Training: Waiting Time (2”), Communicative Turn and Eye Contact:
	● To develop skills that promote good communication, promoting self-confidence and security, integrating them with smoothing (Communicative Skills)	o Picture naming;
		o Reading word lists looking in the mirror and looking at the therapist.
10	● To facilitate voice production	● Body relaxation;
	● To understand how fluent speech and stuttering works (Awareness)	● Breathing exercises;
		● Orofacial motricity exercises;
	● To learn and use smooth and continuous articulatory contact point (Fluency Modeling)	● Communicative Skills Training: Waiting Time (2”), Communicative Turn and Eye Contact:
	● To develop skills that promote good communication, promoting self-confidence and security, integrating them with smoothing (Communicative Skills)	o Own name;
		o Other names, other naming and *brainstorming*;
		o Repetition of sentences looking in the mirror and looking at the therapist.
11	● To facilitate voice production	● Body relaxation;
	● To understand how fluent speech and stuttering works (Awareness)	● Breathing exercises;
		● Orofacial motricity exercises;
	● To accept the presence of stuttering and learn to use strategies to change it (Stuttering Modification)	● Volunteer Stuttering Training:
		o Picture naming;
		o Own name;
		o Formulation of sentences.
12	● To facilitate voice production	● Body relaxation;
	● To understand how fluent speech works (Awareness)	● Breathing exercises;
		● Orofacial motricity exercises;
	● To learn and use smooth and continuous articulatory contact point (Fluency Modeling)	● Communicative Skills Training: Waiting Time (2”), Communicative Turn and Eye Contact:
	● To develop skills that promote good communication, promoting self-confidence and security, integrating them with smoothing (Communicative Skills)	o Other names, other naming and *brainstorming*;
		o Repetition of sentences looking in the mirror and looking at the therapist;
		o Description of the characteristics, parts and functions of objects.
	● To accept the presence of stuttering and learn to use strategies to change it (Stuttering Modification)	● Volunteer Stuttering Training:
		● Picture naming;
13	● To facilitate voice production	● Mouth relaxation;
	● To understand how fluent speech works (Awareness)	● Breathing exercises;
		● Orofacial motricity exercises;
	● To accept the presence of stuttering and learn to use strategies to change it (Stuttering Modification)	● Volunteer Stuttering:
		o Picture naming;
		o Own name;
		o Formulation of sentences;
		o Repetition of sentences looking in the mirror and looking at the therapist.
14	● To facilitate voice production	● Mouth relaxation;
	● To understand how fluent speech works (Awareness)	● Breathing exercises;
		● Orofacial motricity exercises;
	● To accept the presence of stuttering and learn to use strategies to change it (Stuttering Modification)	● Cancellation and Skipping
		o Picture naming;
		o Own name;
		o Reading word lists looking in the mirror and looking at the therapist.
15 16, 17,18 and 19	● To facilitate voice production	● Mouth relaxation;
	● To understand how fluent speech works (Awareness)	● Breathing exercises;
		● Orofacial motricity exercises;
	● To use stuttering modification techniques associated with skills that promote good communication, integrating them with smoothing (Fluency Modeling, Stuttering Modification and Communicative Skills)	● Waiting Time Training (2”), Communicative Turn, Eye Contact and Cancellation and Skipping Techniques:
		o Picture naming;
		o Own name;
		o Reading word lists looking in the mirror and looking at the therapist.
		o Formulation of sentences.
20, 21 and 22	● To facilitate voice production	● Mouth relaxation;
	● To understand how fluent speech works (Awareness)	● Breathing exercises;
		● Orofacial motricity exercises;
	● To use stuttering modification techniques associated with skills that promote good communication, integrating them with smoothing (Fluency Modeling, Stuttering Modification and Communicative Skills)	● Communication Skills Training and Cancellation and Skipping Techniques;
	● Knowing and reducing negative emotions (Desensitization)	● Role-playing everyday situations;
23	● To facilitate voice production	● Breathing Control;
	● To maintain and use the strategies and fluency patterns obtained in previous sessions	● Speech Training:
		o Reduction in the number of breaks per minute;
		● Perceiving the fluency;
		● Dealing with speech:
		o Aspects to remember;
		o Speech diary;
		o Warning the listener.
24	● To facilitate voice production	● Breathing Control;
	● To maintain and use the strategies and fluency patterns obtained in previous sessions	● Speech Training:
		o Reduction in the number of breaks per minute;
		● Perceiving the fluency;
		● Controlling negative thoughts:
		o Bob and Ken story;
		o How to avoid negative thoughts;
		o Change Table.
25	● To maintain and use the strategies and fluency patterns obtained in previous sessions	● Speech Training:
		o Reduction in the number of stuttering episodes per 5 minutes;
		o Speech pause.
		● Keeping the fluency:
		o What helps maintain fluency;
		o How to deal with speech before, during and after stuttering.
		● Managing negative stress:
		o Keeping a calm attitude;
		o Looking after the environment;
		o Taking care of comfort;
		o Taking care of the health.
26	● To maintain and use the strategies and fluency patterns obtained in previous sessions	● Speech Training:
		o Reduction in the number of stuttering episodes per 5 minutes;
		o Speech pause.
		● Facing the phone training in therapy:
		o Phone call simulation.
27	● To maintain and use the strategies and fluency patterns obtained in previous sessions	● Speech Training:
		o Reduction in the number of stuttering episodes in the conversation;
		o Speech rate control.
		● Facing the phone:
		o Phone call to acquaintances.
28	● To maintain and use the strategies and fluency patterns obtained in previous sessions	● Speech Training:
		o Reduction in the number of stuttering episodes in the conversation;
		o Speech rate control.
		● How to control tension;
		o Alone
		o While standing in a public place;
		o While sitting in public places.
		● Facing the phone:
		o Phone call to acquaintances;
		o Phone call to strangers.
29	● To maintain and use the strategies and fluency patterns obtained in previous sessions	● Simulation of tension control:
		o Alone
		o While standing in a public place;
		o While sitting in public places.
		● Conversation about preventing relapses in speech control;
		● Facing the phone:
		o Phone call to acquaintances;
		o Phone call to strangers.
30	● To collect data for evaluation after intensive care	● Speech Sample Collection;
		● OASES-A questionnaire application;
		● Final guidance;
		● Analysis of speech samples of the patient pre- and post-therapy:
		● Self-assessment.

Source: Prepared by the authors based on strategies from PFPF^(8)^ and Fluency Workshop^(9)^

The reassessment was performed after 30 hours of therapy, using the same assessment instruments. Thus, this study will present the data referring to the speech samples and obtained in the application of the OASES-A from before and after undergoing intensive speech-language pathology therapy. The analysis of speech samples was performed by two evaluators, who were experts in Fluency. The number of syllables in each sample ranged from 210 to 232 syllables, except for the sample of a more severe participant, who did not include the 200 syllables proposed by the author of the PAPF, presenting 67 and who presented 67 and 147 syllables in the pre- and post-therapy samples, respectively.

Descriptive data analysis was performed through the frequency distribution of categorical variables and analysis of measures of central tendency and dispersion of continuous variables. The agreement between the evaluations performed by the two evaluators was verified using the intraclass correlation coefficient (ICC). In this sense, the following results were considered to assess agreement: Insignificant if <0; Weak = 0.00-0.20; Fair = 0.21-0.40; Moderate = 0.41-0.60; Strong = 0.61-0.80; Almost Perfect = 0.81-1.00; and Perfect = 1.00. The researchers also used Spearman's rank correlation coefficient to perform the correlation analysis between the speech sample variables (percentage of speech discontinuity, percentage of stuttering disfluencies, and number of words and syllables per minute) and the OASES- A (degree of impact of each of the 4 parts and total). In this context, the evaluation of the magnitude of the correlation adopted the following parameters: Weak = 0.0-0.4; Moderate = 0.4-0.7 and Strong = 0.7-1.0; provided that the value of p≤0.05. Finally, the researchers used SPSS v25.0 software for data entry, processing and analysis.

## RESULTS

The mean age of the sample was 26 years (Median=26.5 SD=4.55), and the four participants were male. Three participants reported no other cases of stuttering in their families (75%). Two participants classified their stuttering, in the clinical history, as moderate (50%), while one (25%) as severe and another (25%) as very severe. With regard to educational level, two participants (50%) had completed higher education, one participant (25%) had incomplete higher education and one (25%) reported having completed high school.

The descriptive analysis of the speech samples of the four participants of the intensive care program shows that there was a decrease in the percentage of speech discontinuity (mean before=43.15% and after=16.76%) and stuttering disfluencies (mean before=30.85% and after=10.61%) after intensive care. On the other hand, there was an increase in the flow of words per minute (mean before=60.10 and after=82.33) and syllables per minute (mean before=119.10 and after=156.55).

When comparing the descriptive analysis of OASES-A pre and post intensive care, there was a decrease in the mean and median in the post therapy assessment in all parts of the questionnaire, as well as in the total score. The findings in the analysis of the degrees of impact of OASES-A, pre and post therapy, show that: Part 1 (General Information on Speech): Improvement in the impact, since, after intensive care, two participants were classified as having mild to moderate impact and the other two as moderate; Part 2 (Reaction to Stuttering): Improvement in the impact, since, after intensive care, two participants were classified as having mild to moderate impact, which had not occurred in the first evaluation; Part 3 (Communication in Everyday Situations): worsening on impact, as the percentage of moderate to severe increased to 50.0% after intensive care, while the percentage of moderate decreased to 25.0%; and Part 4 (Quality of Life): Improvement in impact after intensive care, as participants were classified as mild to moderate (50.0%) and no participants were classified as moderate to severe. The degree of total impact had the same result as in part 4. Table 1 summarizes the results of the analysis of speech samples and the degree of impact obtained by the OAES-A, by participant.

**Table 1:** Descriptive data on fluency and degree of impact of stuttering before and after intensive care, by participant

**Variables**	**Pre-Therapy PAPF**	**Post-Therapy PAPF**	**Pre-Therapy OASES**	**Post-Therapy OASES**
**Participant #1**				
Percentage of speech discontinuity	95.5%	39.4%	Grade 4	Grade 3
Percentage of stuttering disfluencies	70.0%	23.1%	Moderate to Severe	Moderate
Speech rate (words per minute)	14.6	37.4		
Speech rate (syllables per minute)	23.9	66.3		
**Participant #2**				
Percentage of speech discontinuity	11.2%	2.3%	Grade 3	Grade 2
Percentage of stuttering disfluencies	3.1%	0.9%	Moderate	Mild to moderate
Speech rate (words per minute)	147	113.0		
Speech rate (syllables per minute)	304	215.0		
**Participant #3**				
Percentage of speech discontinuity	36.6%	23.0%	Grade 3	Grade 2
Percentage of stuttering disfluencies	27.6%	18.0%	Moderate	Mild to moderate
Speech rate (words per minute)	30.1	40.2		
Speech rate (syllables per minute)	57.5	77.9		
**Participant #4**				
Percentage of speech discontinuity	29.3%	2.3%	Grade 4	Grade 3
Percentage of stuttering disfluencies	22.7%	0.4%	Moderate to Severe	Moderate
Speech rate (words per minute)	48.7	138.7		
Speech rate (syllables per minute)	91.0	267.0		

Then, correlation analyzes were performed between the variables of the analysis of speech samples and the OASES-A scores in the pre- and post-therapy moments. There was no correlation with statistical significance in any of the variables analyzed - p-value>0.05.

In turn, there was strong agreement in all items analyzed (greater than 0.900) in the assessment of agreement between evaluators.

## DISCUSSION

The four study participants are male, corroborating other studies^(1-3)^ that also reported a higher prevalence of stuttering in males. Three participants reported that they did not have other cases of stuttering in family members, which is in line with the literature that reports that most cases have a genetic origin^(2)^, although there are reports of cases with other origins. It is inferred that the participants may be unaware of cases in their families, probably due to the possibility of remission of stuttering in childhood. In addition, stuttering is a multidimensional disorder that can be impacted by multiple factors, such as pre-peri-postnatal history, family history, environmental factor, and linguistic and cognitive abilities of the individual^(3)^.

Regarding the results presented regarding the analysis of the speech samples before and after therapy, there was an improvement in the fluency profile - with a decrease in the percentage of speech discontinuity and stuttering disfluencies of all participants after intensive care. The findings show that the reduction of disfluencies led to an increase in the flow of words and syllables per minute in three of the four participants. It should be noted that the only participant whose flow of words and syllables per minute decreased had values higher than expected before intensive care^(8)^ Therefore, these results corroborate the therapeutic benefits of the intensive care program. Other studies^(5-7)^ found in the literature also reported a significant decrease in disfluencies after intensive treatment, in addition to finding a significant decrease in the duration (in seconds) of disfluencies and an increase in speech flow after treatment^(11,12)^. It is worth mentioning that the data were obtained immediately before and after intensive care, with no time to identify the effects of generalization or use of the acquired therapeutic strategies on the participant's daily life. The semi-annual follow-up of the participants for an extended period was proposed in order to observe in another study whether the effects of intensive care were maintained and consolidated.

The description of the results of the OASES-A pre and post therapy show that there was an improvement in the degree of impact on the participants' lives after intensive care. This finding is in line with the literature^(12,13)^, which reports that the impact of stuttering is directly related to quality of life in adults who stutter. In this sense, it is inferred that the intervention resulted in better knowledge about stuttering, awareness of the body and speech and perception of the individual's feelings regarding their verbal production, assuming the proposals of awareness and modification of stuttering^(8,9)^.

Regarding the correlation analyzes between the variables of the speech samples and the OASES-A scores in the pre- and post-therapy moments, there was no correlation with statistical significance in any of the analyzed variables. This finding may be related to the time when the questionnaire response was collected, which was immediately after the last therapy session. In this context, the time for the perception of the acquired skills and use of the strategies learned may not have been enough for the participants to perceive the changes caused by the therapy. In addition, the reduced number of participants may also have influenced the analysis. There are many cases in clinical practice in which the self-perception of stuttering and its impact on the individual's life is not proportional to the analysis of the individual's stuttering when performed by the interlocutor. This means that an individual with a mild stutter can have a much more negative impact on communicative experiences than an individual with a severe stutter, and vice versa^(14)^.

Finally, the analysis of agreement between the evaluators shows a strong agreement in the items analyzed pre and post therapy, which indicates that the results obtained in the study are considered reliable.

In turn, the small number of participants may be a limitation of the study, which is due to the difficulty in performing speech-language pathology therapy on a daily basis. Intensive care involves socioeconomic factors, in addition to demanding longitudinal, daily follow-up, which makes it difficult to perform. In this sense, some factors such as availability of time and daily mobility are complicating factors for those interested in intensive care. On the other hand, this intervention can be an excellent strategy given the small number of specialists in the field of Fluency in the country, which compromises the feasibility of adequate treatment for all people who stutter, and who live far from places where professionals with expertise in the area work. The intensive care solution could provide adequate care for people who stutter, with intensive treatment in a shorter period of time, such as a vacation away from their hometowns.

This study provides contributions to Speech-Language Pathology so that individuals who stutter can improve speech fluency in a shorter period of time. The study findings were relevant to identify the benefits of intensive care in developmental stuttering and to present the therapeutic model used. The researchers decided to use a mixed model addressing strategies to promote fluency, modify stuttering and improve communicative skills, providing a reduction in the number of disfluencies and an increase in the flow of speech. Finally, the researchers recommend that the study be replicated in a larger sample of adults who stutter and that other intervention proposals can be made based on these findings, since although positive changes were observed, some results analyzed were not statistically significant.

## CONCLUSION

Given that there was an improvement in speech fluency, a reduction in the percentages of speech discontinuity of stuttering disfluencies, in addition to a reduction in the impact of stuttering on the lives of the participants, which suggests the relevance of the proposal of intensive speech-language pathology therapy, it is possible to conclude that there was an improvement of all variables assessed after intensive care. The findings detail the content of an intensive speech-language pathology therapy program for stuttering, thus allowing other speech-language pathologists to use the proposed intervention. It should be noted that the improvement in fluency and impact of stuttering after intensive speech-language pathology therapy in adults who stutter was verified by descriptive and concordance analyses.
