# Supplementary material for: Intensive speech-language pathology therapy with adults who stutter: preliminary study
Source: Codas. 2023 May 26;35(3):e20210159. doi: 10.1590/2317-1782/20232021159en (PMC10266798; doi:10.1590/2317-1782/20232021159en)
Supplement: Translation

# Translation: Terapia fonoaudiológica intensiva com adultos que gaguejam: estudo preliminar

**DOI:** 10.1590/2317-1782/20232021159pt

**Authors:** Amanda Caroline Pereira da Silva Miranda, Camila Queiroz de Moraes Silveira Di Ninno, Denise Brandão de Oliveira e Britto

**Affiliations:** 1 Departamento de Fonoaudiologia, Faculdade de Medicina, Universidade Federal de Minas Gerais - UFMG - Belo Horizonte (MG), Brasil.; 2 Fonoaudióloga Clínica, Consultório Particular - São Paulo (SP), Brasil.

**Keywords:** Fonoaudiologia, Linguagem, Transtornos da Linguagem, Transtorno da Fluência com Início na Infância, Gagueira

## Abstract

**Objetivo:**

Comparar os resultados da fluência e da autopercepção do impacto da gagueira na vida de adultos que gaguejam, antes e depois de terapia fonoaudiológica intensiva.

**Método:**

Trata-se de estudo descritivo longitudinal com coleta de dados antes e após terapia fonoaudiológica intensiva de quatro pacientes com gagueira. O programa de terapia intensiva constituiu-se em trinta encontros, de uma hora cada, realizados em cinco sessões individuais na semana. As amostras de fala coletadas antes e após a terapia foram analisadas por dois juízes especialistas em Fluência. A análise descritiva dos dados foi realizada por meio da distribuição de frequência das variáveis categóricas e análise das medidas de tendência central e de dispersão das variáveis contínuas. A verificação da concordância entre as análises realizadas pelos dois juízes foi realizada por meio do coeficiente de correlação intraclasse (CCI). Foi realizada também a análise de correlação pelo coeficiente de correlação de Spearman, entre as variáveis da amostra de fala e os escores do OASES-A.

**Resultados:**

Houve redução do percentual de descontinuidade da fala e do percentual de disfluências gagas, aumentando o fluxo de palavras por minuto dos participantes. Na análise descritiva do OASES-A observou-se que em todas as partes do questionário, houve diminuição do grau de impacto da gagueira na vida dos participantes.

**Conclusão:**

Verificou-se melhora de todas variáveis analisadas após terapia intensiva. Observou-se melhora na fluência da fala e redução do impacto da gagueira na vida dos participantes, sugerindo a relevância da proposta de terapia fonoaudiológica intensiva na gagueira.

## INTRODUÇÃO

A gagueira do desenvolvimento é um transtorno da fluência definido pela presença de rupturas involuntárias do fluxo da fala, caracterizadas por repetições de sons, sílabas e palavras monossilábicas, prolongamentos de sons, bloqueios, pausas extensas e intrusões que interrompem o fluxo contínuo e suave da fala^(1)^. Pode ser definida como resultado de uma disfunção do sistema nervoso central, com base genética, que aparece no período de desenvolvimento da linguagem, entre 18 meses e sete anos de idade. Em 20% dos casos o distúrbio torna-se crônico, resultando em uma prevalência de 1% na idade adulta, com maior ocorrência no sexo masculino^(2)^.

A gagueira é um transtorno do desenvolvimento de aspecto multidimensional, na qual fatores como histórico familial, ambiente, capacidades linguísticas e cognitivas do indivíduo podem interferir^(3)^. Embora a etiologia da gagueira ainda não tenha sido precisamente identificada, pesquisas^(2,3)^ têm mostrado que fatores genéticos estão envolvidos na suscetibilidade do transtorno.

A intervenção fonoaudiológica é imprescindível em indivíduos com gagueira, uma vez que o objetivo do tratamento é a promoção da fluência e redução das disfluências, propiciando maior fluxo de informação e fala contínua e suave, o mais natural possível, tanto para o falante como para o ouvinte^(4)^.

Tradicionalmente, a terapia fonoaudiológica é realizada com um a dois atendimentos semanais. Existem propostas^(5-7)^ afirmando que programas intensivos são uma alternativa para se conseguir um padrão de fala mais fluente em menor tempo. A proposta de terapia intensiva para fluência baseia-se nos modelos norte-americanos, canadenses e europeus de programas terapêuticos para estimulação da linguagem oral, fala e/ou fluência. Possui como característica encontros diários, individuais ou em grupo, em torno de 30 dias consecutivos. Pressupõe-se que a terapia diária, pela proximidade e frequência dos encontros, possibilita a observação gradativa das modificações nos comportamentos comunicativos dos pacientes, auxiliando-os na percepção de suas dificuldades e no uso funcional da linguagem. A terapia intensiva pode ser um módulo inicial da terapia convencional ou uma proposta integral de intervenção. Os programas fonoaudiológicos de intervenção em adultos, tanto intensiva quanto convencional, geralmente apresentam uma abordagem mista, envolvendo estratégias de promoção da fluência, modificação da gagueira e aprimoramento de habilidades comunicativas^(8,9)^.

O presente estudo teve por objetivo comparar os resultados da fluência e da autopercepção do impacto da gagueira na vida de adultos que gaguejam antes e depois de terapia fonoaudiológica intensiva.

## MÉTODO

Trata-se de um estudo preliminar descritivo longitudinal, do tipo experimental e de caráter quantitativo que analisou os resultados da terapia fonoaudiológica intensiva em quatro pacientes do sexo masculino entre 20 e 31 anos de idade, que gaguejam. O estudo foi aprovado pelo Comitê de Ética em Pesquisa da UFMG, por meio do protocolo **CAAE:** 02470618.1.0000.5149.

Os participantes foram recrutados por meio de convite à população que gagueja de Belo Horizonte. Como critério de inclusão foi adotada a disponibilidade em participar da intervenção intensiva (cinco horas/semanais), assinatura do Termo de Consentimento Livre e Esclarecido e queixa de gagueira confirmada pelo percentual de no mínimo 3% de disfluências gagas, na análise da amostra de fala^(8)^. Os critérios de exclusão foram: comprometimento cognitivo, psicológico, neurológico, outros transtornos de linguagem e do neurodesenvolvimento associados (autorreferidos pelos participantes) e ter realizado tratamento para gagueira no último ano. O estudo foi realizado no Ambulatório de Fonoaudiologia do Hospital São Geraldo, complexo do Hospital das Clínicas da UFMG. Os instrumentos e procedimentos de coleta de dados foram: História Clínica; Questionário sobre a gagueira; Protocolo de avaliação do perfil da fluência (PAPF)^(8)^, Instrumento Overall Assessment of the Speaker’s Experience of Stuttering - Adults (OASES-A), traduzido para o Português Brasileiro^(10)^ e programa de terapia intensiva para gagueira elaborado pelas autoras, baseado na literatura^(8,9)^.

Inicialmente, foi levantada a história clínica do paciente por meio da coleta de informações gerais, histórico de gagueira na família, histórico de saúde geral e de problemas fonoaudiológicos, conhecimento geral do participante sobre gagueira, principais fatores associados, início e gravidade da gagueira, sentimentos relacionados à gagueira, impacto da gagueira nas atividades de vida diária e expectativas em relação ao tratamento.

Para realizar o protocolo de avaliação do perfil da fluência da fala^(8)^, obteve-se o registro em áudio e vídeo de amostra de fala encadeada espontânea dos participantes por meio da apresentação pessoal e descrição de figura temática, antes e após as 30 horas de terapia fonoaudiológica.

A aplicação do OASES-A^(10)^ foi realizada antes do início da terapia e na última sessão de terapia. O instrumento é organizado em quatro seções, e cada seção abrange uma temática diferente: Informações Gerais sobre a Fala, Reação à Gagueira, Comunicação em Situações Cotidianas e Qualidade de Vida. Os dados foram analisados com base nos referenciais teóricos que respaldam o instrumento. O escore por seção e o escore global são obtidos pela soma dos escores das quatro seções do instrumento. Na interpretação do resultado da avaliação, o impacto da gagueira sobre o falante pode ser considerado como leve, leve a moderado, moderado, moderado a severo ou severo.

O programa de terapia intensiva constituiu-se em trinta sessões de terapia, realizadas em cinco encontros individuais na semana, totalizando um período de cerca de dois meses de atendimentos. A proposta (Quadro 1) foi elaborada baseada no programa fonoaudiológico de intervenção de promoção da fluência, com estratégias de conscientização, dessensibilização e modelagem da fluência, além de atividades de modificação da gagueira e de aprimoramento de habilidades comunicativas^(8,9)^.

**Quadro 1:** Objetivos e estratégias da intervenção - Programa de terapia intensiva na gagueira

**Sessões**	**Objetivos**	**Estratégias**
1	● Facilitar a emissão	● Relaxamento corporal;
	● Compreender o funcionamento da fala fluente e da gagueira (Conscientização)	● Exercícios para respiração;
		● Exercícios de motricidade orofacial;
		● Definição da Gagueira;
		● O que ajuda e o que atrapalha na gagueira;
	● Aprender a fazer ponto de contato articulatório suave e contínuo (Modelagem da fluência)	● Conceituação Fala Difícil e Fala Fácil;
		● Características da fala e prática negativa (100% tensão, 50% tensão, e suavizada) com bola: nomeação de figuras
2	● Facilitar a emissão	● Relaxamento corporal;
	● Compreender o funcionamento da fala fluente e da gagueira	● Exercícios para respiração;
	● Desmistificar a gagueira e obter informações atualizadas e científicas	● Exercícios de motricidade orofacial;
	● Perceber diferentes maneiras de produção de fala (Conscientização)	● Dinâmica: Máquina de Fala;
		● Dinâmica: Máquina falhando
		● Mitos e Verdades sobre Gagueira
	● Aprender a fazer e usar ponto de contato articulatório suave e contínuo (Modelagem da fluência)	● Características da fala e prática negativa (100%, 50%, e suavizada) com bola:
		o Nomeação de figura;
		● Leitura de listas de palavras olhando para o espelho e olhando para a terapeuta.
3	● Facilitar a emissão	● Relaxamento corporal;
	● Compreender o funcionamento da fala fluente e da gagueira	● Exercícios para respiração;
	● Perceber diferentes maneiras de produção de fala (Conscientização)	● Exercícios de motricidade orofacial;
		● Dinâmica: Fala Maluca;
	● Aprender a fazer e usar ponto de contato articulatório suave e contínuo (Modelagem da fluência)	● Características da fala e prática negativa (100%, 50%, e suavizada) com bola:
		o Nome próprio;
		o Outros nomes, outras nomeações e *brainstorm.*
4	● Facilitar a emissão	● Relaxamento corporal;
	● Compreender o funcionamento da fala fluente e da gagueira	● Exercícios para respiração;
	● Desmistificar a gagueira e obter informações atualizadas e científicas (Conscientização)	● Exercícios de motricidade orofacial;
		● Mitos e Verdades sobre Gagueira;
	● Conhecer e reduzir as emoções negativas (Dessensibilização)	● Dinâmica: Iceberg da Gagueira
	● Aprender a fazer e usar ponto de contato articulatório suave e contínuo (Modelagem da fluência)	● Características da fala e prática negativa (100%, 50%, e suavizada) com bola:
		● Outros nomes, outras nomeações e *brainstorm.*
5	● Facilitar a emissão	● Relaxamento corporal;
	● Compreender o funcionamento da fala fluente e da gagueira	● Exercícios para respiração;
	● Perceber pontos de tensão (Conscientização)	● Exercícios de motricidade orofacial;
		● Percepção dos pontos de tensão da fala;
	● Aprender a fazer e usar ponto de contato articulatório suave e contínuo (Modelagem da fluência)	● Percepção da fala fácil / fala normal / fala difícil;
		● Características da fala e prática negativa (suavizada) com mola:
		● Repetição de frases.
6	● Facilitar a emissão	● Relaxamento corporal;
	● Compreender o funcionamento da fala fluente e da gagueira (Conscientização)	● Exercícios para respiração;
		● Exercícios de motricidade orofacial;
	● Conhecer e reduzir as emoções negativas (Dessensibilização)	● Pensamentos bons e maus relacionados à gagueira;
	● Aprender e usar ponto de contato articulatório suave e contínuo (Modelagem da fluência)	● Características da fala e prática negativa (suavizada) com mola:
		o Repetição de frases;
		o Leitura de frases olhando para o espelho e olhando para a terapeuta.
7	● Facilitar a emissão	● Relaxamento corporal;
	● Compreender o funcionamento da fala fluente e da gagueira (Conscientização)	● Exercícios para respiração;
		● Exercícios de motricidade orofacial;
	● Conhecer e reduzir as emoções negativas (Dessensibilização)	● Dramatizações de situações cotidianas;
	● Aprender e usar ponto de contato articulatório suave e contínuo (Modelagem da fluência)	● Características da fala e prática negativa (suavizada) com mola:
		o Leitura de frases olhando para o espelho e olhando para a terapeuta;
		o Completar frases prontas com figuras do baralho;
		o Elaboração de frases espontâneas com figuras do baralho.
		o Treino de fala encadeada com linha.
8	● Facilitar a emissão	● Relaxamento corporal;
	● Compreender o funcionamento da fala fluente e da gagueira (Conscientização)	● Exercícios para respiração;
		● Exercícios de motricidade orofacial;
	● Aprender e usar ponto de contato articulatório suave e contínuo (Modelagem da fluência)	● Características da fala e prática negativa (suavizada) com mola;
		o Completar frases prontas com figuras do baralho;
		o Elaboração de frases espontâneas com figuras do baralho.
		o Dramatização de diálogos com a linha.
9	● Facilitar a emissão	● Relaxamento corporal;
	● Compreender o funcionamento da fala fluente e da gagueira (Conscientização)	● Exercícios para respiração;
		● Exercícios de motricidade orofacial;
	
	● Aprender e usar ponto de contato articulatório suave e contínuo (Modelagem da fluência)	● Treino de Habilidades Comunicativas: Tempo de Espera (2”), Turno Comunicativo e Contato de Olhos:
	● Desenvolver habilidades que promovem a boa comunicação, promovendo autoconfiança e segurança, integrando-as à suavização (Habilidades Comunicativas)	o Nomeação de figuras;
		o Leitura de listas de palavras olhando para o espelho e olhando para a terapeuta.
10	● Facilitar a emissão	● Relaxamento corporal;
	● Compreender o funcionamento da fala fluente e da gagueira (Conscientização)	● Exercícios para respiração;
		● Exercícios de motricidade orofacial;
	● Aprender e usar ponto de contato articulatório suave e contínuo (Modelagem da fluência)	● Treino de Habilidades Comunicativas: Tempo de Espera (2”), Turno Comunicativo e Contato de Olhos:
	● Desenvolver habilidades que promovem a boa comunicação, promovendo autoconfiança e segurança, integrando-as à suavização (Habilidades Comunicativas)	o Nome próprio;
		o Outros nomes, outras nomeações e *brainstorm;*
		o Repetição de frases olhando para o espelho e olhando para a terapeuta.
11	● Facilitar a emissão	● Relaxamento corporal;
	● Compreender o funcionamento da fala fluente e da gagueira (Conscientização)	● Exercícios para respiração;
		● Exercícios de motricidade orofacial;
	● Aceitar que a gagueira acontece e aprender a utilizar estratégias para modifica-la (Modificação da Gagueira)	● Treino Gagueira Voluntária:
		o Nomeação de figuras;
		o Nome próprio;
		o Formulação de frases.
12	● Facilitar a emissão	● Relaxamento corporal;
	● Compreender o funcionamento da fala fluente (Conscientização)	● Exercícios para respiração;
		● Exercícios de motricidade orofacial;
	● Aprender e usar ponto de contato articulatório suave e contínuo (Modelagem da fluência)	● Treino de Habilidades Comunicativas: Tempo de Espera (2”), Turno Comunicativo e Contato de Olhos:
	● Desenvolver habilidades que promovem a boa comunicação, promovendo autoconfiança e segurança, integrando-as à suavização (Habilidades Comunicativas)	o Outros nomes, outras nomeações e *brainstorm;*
		o Repetição de frases olhando para o espelho e olhando para a terapeuta;
		o Descrição de características, partes e funções dos objetos.
	● Aceitar que a gagueira acontece e aprender a utilizar estratégias para modifica-la (Modificação da Gagueira)	● Treino Gagueira Voluntária:
		● Nomeação de figuras.
13	● Facilitar a emissão	● Relaxamento da boca;
	● Compreender o funcionamento da fala fluente (Conscientização)	● Exercícios para respiração;
		● Exercícios de motricidade orofacial;
	● Aceitar que a gagueira acontece e aprender a utilizar estratégias para modifica-la (Modificação da Gagueira)	● Gagueira Voluntária:
		o Nomeação de figuras;
		o Nome próprio;
		o Formulação de frases;
		o Repetição de frases olhando para o espelho e olhando para a terapeuta.
14	● Facilitar a emissão	● Relaxamento da boca;
	● Compreender o funcionamento da fala fluente (Conscientização)	● Exercícios para respiração;
		● Exercícios de motricidade orofacial;
	● Aceitar que a gagueira acontece e aprender a utilizar estratégias para modifica-la (Modificação da Gagueira)	● Cancelamento e Pulando Fora
		o Nomeação de figuras;
		o Nome Próprio;
		o Leitura de listas de palavras olhando para o espelho e olhando para a terapeuta.
15 16, 17,18 e 19	● Facilitar a emissão	● Relaxamento da boca;
	● Compreender o funcionamento da fala fluente (Conscientização)	● Exercícios para respiração;
		● Exercícios de motricidade orofacial;
	● Utilizar técnicas de modificação da gagueira associadas às habilidades que promovem a boa comunicação, integrando-as à suavização (Modelagem da fluência, Modificação da Gagueira e Habilidades Comunicativas)	● Treino de Tempo de Espera (2”), Turno Comunicativo, Contato de Olhos e técnica de Cancelamento e Pulando Fora:
		o Nomeação de figuras;
		o Nome Próprio;
		o Leitura de listas de palavras olhando para o espelho e olhando para a terapeuta.
		o Formulação de frases.
20, 21 e 22	● Facilitar a emissão	● Relaxamento da boca;
	● Compreender o funcionamento da fala fluente (Conscientização)	● Exercícios para respiração;
		● Exercícios de motricidade orofacial;
	● Utilizar técnicas de modificação da gagueira associadas às habilidades que promovem a boa comunicação, integrando-as à suavização (Modelagem da fluência, Modificação da Gagueira e Habilidades Comunicativas)	● Treino de habilidades comunicativas e técnica de Cancelamento e Pulando Fora:
	● Conhecer e reduzir as emoções negativas (Dessensibilização)	● Dramatizações de situações cotidianas.
23	● Facilitar a emissão	● Controle Respiratório;
	● Manter e utilizar as estratégias e os padrões de fluência obtidos nas sessões anteriores	● Treino de fala:
		o Redução do número de rupturas por minuto;
		● Percebendo a fluência;
		● Lidando com a fala:
		o Pontos a serem lembrados;
		o Diário de fala;
		o Avisando o ouvinte.
24	● Facilitar a emissão	● Controle Respiratório;
	● Manter e utilizar as estratégias e os padrões de fluência obtidos nas sessões anteriores	● Treino de fala:
		o Redução do número de rupturas por minuto;
		● Percebendo a fluência;
		● Controlando pensamentos negativos:
		o História de Bob e Ken;
		o Como evitar pensamentos negativos;
		o Tabela da Mudança.
25	● Manter e utilizar as estratégias e os padrões de fluência obtidos nas sessões anteriores	● Treino de fala:
		o Redução do número de gagueira por 5 minutos;
		o Pausamento da fala.
		● Mantendo a fluência:
		o O que ajuda a manter a fluência;
		o Como lidar com a fala antes, durante e depois da gagueira.
		● Controlando o stress negativo:
		o Manter atitude calma;
		o Cuidar do ambiente;
		o Cuidar do conforto;
		o Cuidar da saúde.
26	● Manter e utilizar as estratégias e os padrões de fluência obtidos nas sessões anteriores	● Treino de fala:
		o Redução do número de gagueira por 5 minutos;
		o Pausamento da fala.
		● Enfrentando o telefone treino em terapia:
		o Simulação de ligação telefônica.
27	● Manter e utilizar as estratégias e os padrões de fluência obtidos nas sessões anteriores	● Treino de fala:
		o Redução do número de gagueira no diálogo;
		o Controle da velocidade de fala.
		● Enfrentando o telefone:
		o Ligação para conhecidos.
28	● Manter e utilizar as estratégias e os padrões de fluência obtidos nas sessões anteriores	● Treino de fala:
		o Redução do número de gagueira no diálogo;
		o Controle da velocidade de fala.
		● Como controlar a tensão;
		o Sozinho;
		o Em pé em local público;
		o Sentado em locais públicos.
		● Enfrentando o telefone:
		o Ligação para conhecidos;
		o Ligação para estranhos.
29	● Manter e utilizar as estratégias e os padrões de fluência obtidos nas sessões anteriores	● Simulação de controle da tensão:
		o Sozinho;
		o Em pé em local público;
		o Sentado em locais públicos.
		● Conversa sobre prevenir recaídas no controle da fala;
		● Enfrentando o telefone:
		o Ligação para conhecidos;
		o Ligação para estranhos.
30	● Coletar dados para avaliação após terapia intensiva	● Coleta de Amostra de Fala;
		● Aplicação questionário OASES-A;
		● Orientações finais;
		● Análise do paciente amostras de fala pré e pós terapia:
		● Auto avaliação.

Fonte: Organizado pelas autoras baseado em estratégias do PFPF^(8)^ e Oficina de Fluência^(9)^

A reavaliação ocorreu após 30 horas de terapia, utilizando os mesmos instrumentos da avaliação. Nesse estudo serão apresentados os dados referentes às amostras de fala e obtidos na aplicação do OASES-A antes e após terapia fonoaudiológica intensiva. As análises das amostras de fala foram realizadas por dois juízes, especialistas em Fluência. O número de sílabas em cada amostra variou de 210 a 232 sílabas, com exceção da amostra de um participante, mais grave, que não contemplou as 200 sílabas propostas pela autora do PAPF, apresentando 67 e 147 sílabas, nas amostras pré e pós terapia, respectivamente.

A análise descritiva dos dados foi realizada por meio da distribuição de frequência das variáveis categóricas e análise das medidas de tendência central e de dispersão das variáveis contínuas. A verificação da concordância entre as análises realizadas pelos dois juízes foi realizada por meio do coeficiente de correlação intraclasse (CCI). A concordância foi considerada insignificante se < 0, fraca = 0,00-0,20, razoável = 0,21-0,40, moderada = 0,41-0,60, forte = 0,61-0,80, quase perfeita = 0,81-1,00, e perfeita = 1,00. Realizou-se também a análise de correlação pelo coeficiente de correlação de Spearman, entre as variáveis da amostra de fala (percentual de descontinuidade de fala, percentual de disfluências gagas e número de palavras e de sílabas por minuto) e os escores do OASES-A (grau de impacto de cada uma das 4 partes e total). Para tal, a magnitude da correlação foi medida seguindo o seguinte parâmetro: fraca = 0,0-0,4; moderada = 0,4-0,7 e forte = 0,7-1,0; desde que com valor de p≤0,05. Para entrada, processamento e análise dos dados utilizou-se o software SPSS, versão 25.0.

## RESULTADOS

A média de idade da amostra foi de 26 anos (Mediana=26,5 DP=4,55), sendo os quatro participantes do sexo masculino. Três participantes não relataram outros casos de gagueira na família (75%). Dois classificaram sua gagueira, na história clínica, como moderada (50%), um (25%) classificou como grave e outro (25%) como muito grave. Quanto à escolaridade, dois participantes (50%) concluíram o curso superior, um participante (25%) superior incompleto e um (25%) relatou ter concluído o ensino médio.

A análise descritiva das amostras de fala dos quatro participantes do programa de terapia intensiva permitiu descrever que o percentual de descontinuidade de fala (Média pré=43,15% e Média após=16,76%) e o percentual de disfluências gagas (Média pré=30,85% e Média após=10,61%) diminuíram após a terapia intensiva. Por outro lado, os fluxos de palavras por minuto (Média pré=60,10 e Média após=82,33) e de sílabas por minuto (Média pré=119,10 e Média após=156,55) aumentaram.

Comparando-se a análise descritiva do OASES-A pré e pós terapia intensiva observa-se que em todas as partes do questionário, bem como em seu escore total, houve diminuição da média e da mediana na avaliação pós terapia. Na análise dos graus de impacto do OASES-A, pré e pós terapia, verificou-se que: Parte 1 (Informações Gerais), melhora do impacto, em que dois participantes do pós foram classificados como impacto leve a moderado e a outros dois como moderado; Parte 2 (Suas reações à gagueira), melhora do impacto, em que dois do pós foram classificados como impacto leve a moderado, o que não havia ocorrido na primeira avaliação; Parte 3 (Comunicação nas situações diárias), piora no impacto em que a porcentagem do moderado a severo do pós aumentou para 50,0%, enquanto o moderado caiu para 25,0%; Parte 4 (Qualidade de vida), melhora no impacto no pós com participantes classificados no grau leve a moderado (50,0%) e ausência de participantes no moderado a severo. O grau de impacto total teve o mesmo resultado da parte 4. A Tabela 1 sintetiza os resultados das análises das amostras de fala e do grau de impacto obtido pelos OAES-A, por participante.

**Tabela 1:** Dados descritivos da fluência e grau de impacto da gagueira antes e depois da terapia intensiva, por participante

**Variáveis**	**PAPF Pré**	**PAPF Pós**	**OASES Pré**	**OASES Pós**
**Participante 1**				
% de descontinuidade de fala	95,5%	39,4%	Grau 4	Grau 3
% de disfluências gagas	70,0%	23,1%	MODERADO A SEVERO	MODERADO
Velocidade de fala (palavras/minuto)	14,6	37,4		
Velocidade de fala (sílabas/minuto)	23,9	66,3		
**Participante 2**				
% de descontinuidade de fala	11,2%	2,3%	Grau 3	Grau 2
% de disfluências gagas	3,1%	0,9%	MODERADO	LEVE A MODERADO
Velocidade de fala (palavras/minuto)	147	113,0		
Velocidade de fala (sílabas/minuto)	304	215,0		
**Participante 3**				
% de descontinuidade de fala	36,6%	23,0%	Grau 3	Grau 2
% de disfluências gagas	27,6%	18,0%	MODERADO	LEVE A MODERADO
Velocidade de fala (palavras/minuto)	30,1	40,2		
Velocidade de fala (sílabas/minuto)	57,5	77,9		
**Participante 4**				
% de descontinuidade de fala	29,3%	2,3%	Grau 4	Grau 3
% de disfluências gagas	22,7%	0,4%	MODERADO A SEVERO	MODERADO
Velocidade de fala (palavras/minuto)	48,7	138,7		
Velocidade de fala (sílabas/minuto)	91,0	267,0		

Em seguida foram realizadas análises de correlação entre as variáveis da análise das amostras de fala e os escores do OASES-A nos momentos pré e pós terapia. Não houve correlação com significância estatística em quaisquer das variáveis analisadas - valor de p>0,05.

Na análise de concordância entre os juízes observou-se que houve concordância forte em todos os itens analisados (maiores que 0,900).

## DISCUSSÃO

Os quatro participantes do estudo são do sexo masculino, corroborando outras investigações^(1-3)^ cujos autores relataram maior prevalência de gagueira no sexo masculino. Três participantes relataram não possuir outros membros na família que gaguejam. Tal fato vai de encontro à literatura que relata que a maioria dos casos tem origem genética^(2)^, embora haja relatos de casos com outras origens. Infere-se que os participantes podem desconhecer casos em suas famílias, provavelmente pela possibilidade de remissão da gagueira na infância. Além disso, a gagueira é um transtorno de aspecto multidimensional, no qual inúmeros fatores podem interferir, como histórico pré-peri-pós-natal, histórico familiar, fator ambiental, capacidades linguísticas e cognitivas do indivíduo^(3)^.

Em relação aos resultados apresentados acerca das análises das amostras de fala pré e pós terapia, observou-se melhora no perfil da fluência - com diminuição da porcentagem de descontinuidade de fala e de disfluências gagas de todos os participantes após terapia intensiva. Os achados indicam que a redução das disfluências, levou ao aumento no fluxo de palavras e sílabas por minuto, em três dos quatro participantes Ressalta-se que o único participante cujo fluxo de palavras e de sílabas por minuto diminuiu apresentava antes da terapia intensiva valores acima do esperado^(8)^. Estes resultados confirmam os benefícios terapêuticos do programa de terapia intensiva. Na literatura, outros estudos^(5-7)^ confirmam diminuição significativa das disfluências após tratamento intensivo. Além disso, apontam diminuição significativa da duração (em segundos) de disfluências e aumento do fluxo de fala após o tratamento^(11,12)^. Vale ressaltar que os dados foram obtidos imediatamente antes e após a terapia intensiva, sem tempo para identificar os efeitos da generalização ou uso das estratégias terapêuticas adquiridas, no dia a dia do participante. O acompanhamento semestral dos participantes por um tempo estendido foi proposto com vistas a observar, em outro estudo, se os efeitos da terapia intensiva foram mantidos e consolidados.

A descrição dos resultados do OASES-A, pré e pós terapia, indicam que após a terapia intensiva houve melhora no grau do impacto na vida dos participantes. Tal achado corrobora a literatura^(12,13)^, que aponta que o impacto da gagueira está diretamente relacionado à qualidade de vida em pessoas adultas que gaguejam. Infere-se que a intervenção resultou em melhor conhecimento sobre a gagueira, conscientização do corpo e da fala e percepção dos sentimentos do sujeito frente a sua produção verbal, assumindo as propostas de conscientização e modificação da gagueira^(8,9)^.

Em relação às análises de correlação entre as variáveis das amostras de fala e os escores do OASES-A nos momentos pré e pós terapia, não houve correlação com significância estatística em quaisquer das variáveis analisadas. Tal fato pode estar relacionado ao momento de resposta do questionário - imediatamente após a última sessão de terapia. O tempo para percepção das habilidades adquiridas e de uso das estratégias aprendidas pode não ter sido suficiente para que os participantes percebessem as modificações ocasionadas com a terapia. O número reduzido de participantes pode, também, ter influenciado a análise. Na prática clínica observa-se que muitas vezes a autopercepção da gagueira e o impacto da mesma na vida do falante não é proporcional à análise da gagueira deste mesmo falante, realizada pelo interlocutor, ou seja, um falante que gagueja levemente pode ter uma interferência muito mais negativa nas experiências comunicativas que um falante com gagueira severa, e vice-versa^(14)^.

Por fim, a análise de concordância entre os juízes aponta para concordância forte nos itens analisados pré e pós terapia, sendo assim, os resultados obtidos no estudo são considerados confiáveis.

Como limitação do estudo considera-se o pequeno número de participantes decorrente da dificuldade em realizar diariamente a terapia fonoaudiológica. A terapia intensiva envolve fatores socioeconômicos, além de demandar o acompanhamento longitudinal, diário, que dificulta a realização. Fatores como disponibilidade de horário e de locomoção diários são complicadores para os interessados na terapia intensiva. Em contrapartida infere-se que seja uma excelente estratégia a ser considerada levando-se em conta o pequeno número de especialistas na área da Fluência no país, comprometendo a viabilidade de tratamento adequado a todas as pessoas que gaguejam, distantes de locais onde atuam profissionais com expertise na área. A terapia intensiva viabiliza que a pessoa que gagueja possa receber atendimento adequado, intensivamente, em um espaço de tempo mais curto, longe da sua cidade de origem, em seu período de férias, por exemplo.

Como avanço, este estudo traz contribuições para a Fonoaudiologia e para que falantes que gaguejam possam melhorar a fluência da fala em um espaço de tempo menor. Os resultados foram importantes para identificar os benefícios da terapia intensiva na gagueira do desenvolvimento e apresentar o modelo terapêutico utilizado. Optou-se por utilizar um modelo misto abordando estratégias de promoção da fluência, modificação da gagueira e aprimoramento das habilidades comunicativas, propiciando a redução no número de disfluências e aumento do fluxo de fala. Sugere-se que esse estudo seja replicado numa amostra maior de adultos que gaguejam e que outras propostas de intervenção possam vir a ser feitas com base nesses achados, uma vez que apesar de mudanças positivas terem sido observadas, alguns resultados analisados não foram estatisticamente significantes.

## CONCLUSÃO

Conclui-se que houve melhora de todas variáveis analisadas após terapia intensiva. Observou-se melhora na fluência da fala, redução dos percentuais de descontinuidade de fala de disfluências gagas, além da redução do impacto da gagueira na vida dos participantes, o que sugere a relevância da proposta de terapia fonoaudiológica intensiva. Os dados expostos possibilitam mostrar o conteúdo de um programa de terapia fonoaudiológica intensiva da gagueira permitindo que outros fonoaudiólogos possam utilizar a intervenção proposta, cuja melhora da fluência e do impacto da gagueira após terapia fonoaudiológica intensiva em adultos que gaguejam foram constatados pelas análises descritivas e de concordância.
